# Distended bladder presenting with constipation and venous obstruction: a case report

**DOI:** 10.1186/1752-1947-6-34

**Published:** 2012-01-24

**Authors:** Anu Sharma, Vijay Naraynsingh

**Affiliations:** 1 Faculty of the Clinical Surgical Sciences, University of the West Indies, St. Augustine, Trinidad & Tobago

## Abstract

**Introduction:**

A distended urinary bladder has been known to cause venous obstruction or rarely bowel obstruction. We report the first case in the literature in which urinary bladder distension presented with both venous obstruction and constipation. This is an unusual presentation of urinary bladder distension and serves to broaden our differential diagnoses for a patient with clinical venous obstruction.

**Case presentation:**

An 83-year-old man of African descent presented with constipation and bilateral lower limb edema. A huge abdominal mass was evident which was a large, distended urinary bladder confirmed by computed tomography. Promptly after urethral catheterization, both constipation and lower limb edema resolved.

**Conclusions:**

To the best of our knowledge distended urinary bladder causing both constipation and lower limb edema has never previously been reported. Analysis of the literature revealed several factors resulting in the patient's presentation. A high level of suspicion for urinary bladder distension must be maintained for prompt diagnosis and to avoid improper management.

## Introduction

Chronically distended urinary bladder is diagnosed in only 0.8% [1] of elderly men annually. A distended urinary bladder causing inferior vena cava and external iliac venous obstruction has been commonly described [2] but is infrequently encountered. Its presentation with bilateral pedal edema poses a clinical dilemma as it can be easily misdiagnosed as deep venous thrombosis or congestive heart failure. Bladder distension causing constipation, however, is very rare with few reports in the literature. To the best of our knowledge and after extensive literature review, we present the first case of chronic bladder distension presenting with both constipation and bilateral pedal edema.

### Case Presentation

An 83-year-old diabetic man of African descent presented with a four week history of constipation and a two week history of obstipation to our emergency department. He noticed painless increasing abdominal distension with concomitant leg edema. He denied any history of vomiting, fever or anorexia. He used several laxative concoctions with no relief. He revealed a long standing history of incontinence at night with hesitancy and poor stream but denied frequency and strangury. He had been diabetic and on treatment for 25 years with a history of diabetic retinopathy.

**Figure 1:**
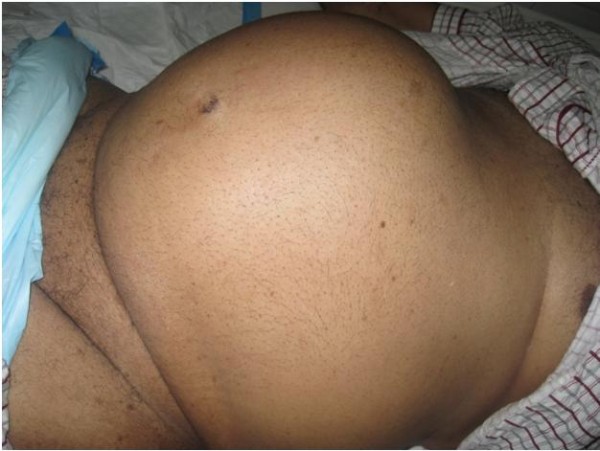
**Large abdominal mass extending to the epigastric region**. The photograph shows the large abdominal mass found on general inspection of the abdomen. It arose out of the pelvis and was felt up to 4 cm below the xiphisternum.

**Figure 2:**
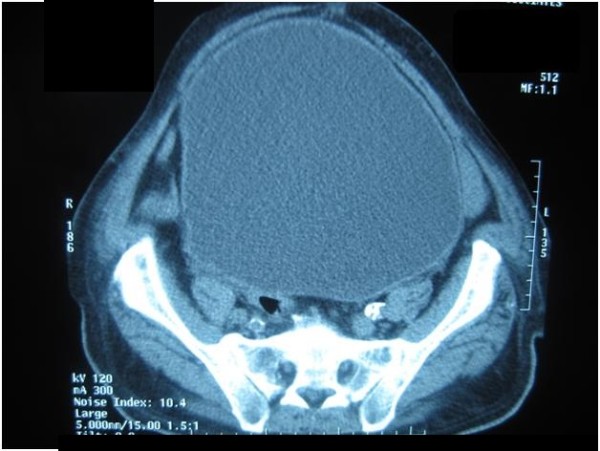
**Computed tomography scan of the abdomen showing a distended urinary bladder**. Computed tomography image of the abdomen found a grossly distended urinary bladder extending out of the pelvis.

**Figure 3:**
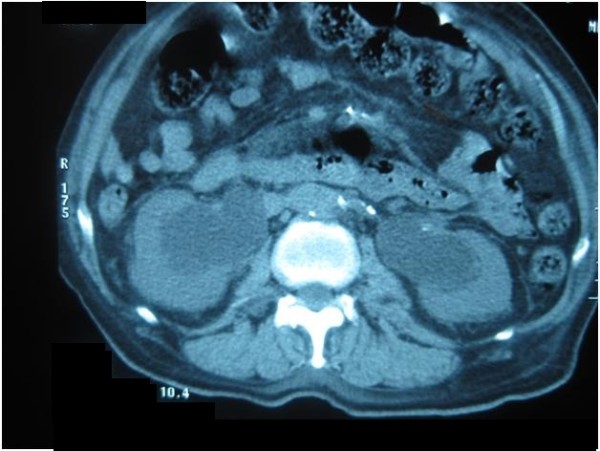
**Computed tomography scan of the abdomen showing bilateral hydronephrosis and hydroureters**. Computed tomography image showing bilateral hydronephrosis and hydroureters due to bladder outlet obstruction. The patient's creatinine was significantly elevated from post-renal failure. After catheterization, creatinine normalized.

On admission, he was tachycardic (pulse 102 beats per minute) with a blood pressure of 160/85 mm Hg and random blood glucose of 511 mg/dL (28.39 mmol/L). He had bilateral pitting edema up to mid leg. A large abdominal mass extended out of the pelvis just below the xiphisternum (Figure 1). The mass was dull to percussion and tender on palpation. His bowel sounds were hyperactive. A symmetrically enlarged, smooth, rubbery prostate was felt on palpation with a bulge above the prostate. All other examinations were normal. Blood test results revealed a normochromic normocytic anemia (hemoglobin 10.4 g/dL, MCV 90.9 fL) with renal impairment (blood urea nitrogen (BUN) 30 mg/dL, creatinine 1.8 mg/dL) and a normal prostate-specific antigen (PSA) level (4 ng/mL). Computed tomography (CT) of the abdomen was ordered with a working diagnosis of bowel obstruction likely secondary to a tumor in mind. The results showed gross distension of the urinary bladder above the level of the umbilicus with marked bilateral hydronephrosis and hydroureter (Figures 2 and 3).

A Foley catheter was inserted which yielded 5000 ml of clear urine. On the following day the patient passed flatus and his leg edema had decreased markedly. He passed stool on the third day with the aid of a suppository. The catheter was removed at the patient's request and he was sent home passing urine freely. His renal impairment resolved (creatinine (Cr) 1.5 mg/dL) on discharge. He was non-compliant with follow up.

One week later, he returned to the emergency department with constipation and bilateral pedal edema. A Foley catheter was re-inserted and 3100 ml of clear urine was drained. The edema completely resolved thereafter and the patient was sent home with the catheter *in situ*.

## Discussion

Due to its fixed volume of the pelvis, bladder distension can compress adjacent structures. It has been known to compress the inferior vena cava [2] and both right and left external iliac veins [3]. In a few cases, this had led to deep vein thrombosis formation [3]. Hopkins *et al. *[4] demonstrated elevated pressures in the femoral veins, more so in cases with urinary retention with bladder capacities more than 1000 ml. Those patients with leg edema were found to have the highest femoral vein pressures. This can account for the lower limb edema found in our case as well as the numerous case reports, albeit small incidence, of venous stasis secondary to bladder distention.

A distended bladder can also compress the recto-sigmoid colon. Kleinhaus *et al. *[5] described four cases of pseudostenosis of the recto-sigmoid colon due to compression by a dilated urinary bladder. Rarely subacute [6] or acute [7] intestinal obstruction can occur. In this case, our patient's clinical presentation was consistent with subacute intestinal obstruction.

In addition to the compressive effect of the distended bladder, constipation would be worsened by the recto-vesicourethral reflex. Buntzen *et al. *[8] showed that anal pressure was markedly increased and rectal motility was absent as bladder distention worsened. This was attributed to the excitatory 'vesico-anal' reflex produced at the spinal cord level. In this case, there was marked bladder distention with a volume of 5000 mLs. Not only was this bladder large enough to compress the colon, but the gross distention would have caused physiological reflex fecal stasis.

Our patient's long standing diabetic status also contributed. Similar to the case presented by Ghebontni *et al. *[6], our patient had long standing diabetes mellitus and prostatic disease. His urinary distension was primarily due to benign prostatic hypertrophy but was significantly worsened by diabetic neuropathy. In evaluating the diabetic neurogenic bladder, it was found that 37.5% [9] of patients had diminished bladder sensation and/or impaired detrusor contractility. In long-standing diabetes mellitus (more than 10 years) significantly reduced basal and anal squeeze pressures and reduced amplitude slow waves were measured [10]. These findings suggest that both the patient's painless bladder distention and constipation could also be attributed to type two diabetes mellitus. The relief of symptoms after catheterization however, suggests that his diabetes played more of an aggravating rather than a causative factor.

## Conclusion

This was an unusual case and first report of a patient presenting primarily for obstipation who was found to have both a clinical subacute intestinal obstruction and lower limb edema due to gross urinary bladder distention aggravated by complicated diabetes mellitus. His urinary symptoms were minimal, leading to his diagnosis by physical and radiological assessments. A high index of suspicion must be maintained for prompt diagnosis and to avoid improper management.

## Consent

Written informed consent was obtained from the patient for publication of this case report and any accompanying images. A copy of the written consent is available for review by the Editor-in-Chief of this journal.

## Competing interests

The authors declare that they have no competing interests.

## Authors' contributions

AS and VN analyzed the patient's data and provided medical assistance to the case. AS performed the literature search and wrote the manuscript. VN edited the manuscript. Both authors read and approved the manuscript.
